# Hygiene knowledge and practices in the Lagos wild meat value chain: Cultural influences, regulatory gaps, and infrastructure needs

**DOI:** 10.1371/journal.pgph.0004321

**Published:** 2026-01-16

**Authors:** Samuel N. Akpan, Elizabeth A. J. Cook, Frank van Langevelde, Pim van Hooft, Dawn M. Zimmerman, Ralph Buij, James M. Hassell, Sherril P. Masudi, Christian T. Happi, Anise N. Happi, Lian F. Thomas

**Affiliations:** 1 Wildlife Ecology and Conservation Group (WEC), Wageningen University and Research, Wageningen, The Netherlands; 2 Institute of Genomics and Global Health (IGH), Redeemers University, Ede, Nigeria; 3 International Livestock Research Institute (ILRI), Nairobi, Kenya; 4 Department of Entomology, Smithsonian Institution–National Museum of Natural History, Washington District of Columbia, United States of America; 5 Department of Epidemiology of Microbial Diseases, Yale School of Public Health, New Haven, Connecticut, United States of America; 6 Animal Ecology Group, Wageningen Environmental Research, Wageningen, The Netherlands; 7 Smithsonian Conservation Biology Institute, Washington District of Columbia, United States of America; 8 Harvard T.H. Chan School of Public Health, Boston, Massachusetts, United States of America; 9 Royal (Dick) School of Veterinary Studies, University of Edinburgh, Midlothian, United Kingdom; Indian Institute of Public Health Shillong, INDIA

## Abstract

Wild meat, commonly known as bushmeat, is a cultural, economic, and nutritional staple food in many regions of the world, including sub-Saharan Africa. Wild meat value chains face major hygiene and sanitary regulation challenges, but only a few studies have investigated these challenges, focusing instead on market dynamics and biodiversity issues. This study examines the hygiene practices, attitudes, perceptions, and knowledge of public health risks among actors in the Lagos (Nigeria) wild meat value chain, and its consequences for food safety. We employed a qualitative study design, using in-depth interviews of key informants (n = 34) purposively selected from the wild meat value chain’s hunter, wholesaler, processor, and retailer nodes. An inductive thematic approach was used for data analysis. Results revealed three overarching themes: culture, infrastructure, and regulation. Social norms, poor infrastructure, and lack of regulation were the main drivers of the hygiene practices in the value chain. Actors showed poor knowledge of the health risks associated with wild meat, prioritizing taste over its safety. Women were more at risk of contracting zoonotic infections due to gender biases, which exposed them to riskier nodes of the value chain. The wild meat value chain in the megacity of Lagos constitutes a high-risk platform for zoonotic and food-borne pathogen transmission, due to poor knowledge, infrastructure deficits, and economic resource pressure, which leads to unhygienic practices of its actors. We recommend intervention approaches that integrate people’s cultures, provision of infrastructure, enforcement of sanitary standards, actors’ education, and further empirical research to stimulate the establishment of hygiene guidelines for the regulation of urban wild meat value chains globally.

## Introduction

Wild meat, commonly referred to as bushmeat, holds significant global cultural importance, including in sub-Saharan Africa [1]. Rapid urbanization and a growing world population have increased the demand for wild meat, putting additional pressure on already fragile ecosystems [2]. Wild meat harvests range from hundreds of thousands of tons per year in Latin America to more than three million tons annually in Central Africa [3,4]. Commercial city centers serve as major hubs for both consumptive use and trafficking of wild meat [5]. Historically, concerns about the wild meat trade have primarily focused on issues related to biodiversity conservation [6], but high-profile disease outbreaks linked to wild meat and contamination of food by wildlife have shifted the focus to public health concerns. For example, in 2017, a deadly outbreak of zoonotic Lassa fever emerged in West Africa [7] that was linked to rodents, particularly the multimammate rat (*Mastomys natalensis*), that played a crucial role by indirectly transmitting the infection to humans through contaminated food products [8]. Also, the Ebola virus disease (EVD) outbreak in the Likati zone of the Democratic Republic of Congo was linked to food via a hunter who had physical contact with fresh wild meats (monkey and wild boar), while the first Lassa fever cases in Ghana’s Ashanti region were traced to persons who had consumed rodents [9,10]. Behavioral practices are one of the key facilitators of zoonotic disease transmission, especially in individuals who have frequent contact with wild animals [11]. Zoonotic disease connected to the bushmeat trade can be directly linked to the primary human risk behaviors: hunting (medium risk), butchering (high risk), and consumption (low risk due to less contact with blood) of infected animals [11]. During hunting activities, for instance, individuals are more susceptible to bites and scratches from infected primates and rodents, particularly if they have open wounds [12]. Poor hygiene practices within wild meat value chains have long been recognized, with low levels of awareness around meat hygiene being particularly evident among wild meat vendors [13]. A study conducted in Kinshasa (Democratic Republic of Congo) revealed that actors expressed belief in transmission of illness from livestock to humans, but not from wild animals to humans [14]. Participants reported that they cut themselves during the process of butchering yet did not consider butchering of wild meats to be a risky activity. Ultimately, wild meats were viewed as pure and natural, in contrast to meats from domestic animals which were considered tainted by human interference [14]. In addition to zoonotic risks, inappropriate handling, processing and storage often lead to meat contamination, which negatively impacts both meat quality and safety, contributing to the transmission of foodborne pathogens [15–17]. Despite these health risks, wild meat trade continues to flourish due to socio-cultural and economic factors [18], exacerbated by inadequate infrastructure and weak regulatory enforcement [19]. Also, food safety regulations are often neglected in developing counties due to low monitoring, lack of awareness and corruption [20], which directly influence sanitation practices.

To support food-borne and zoonotic disease risk mitigation strategies, it is imperative to deepen understanding of the hygiene and handling practices within the wild meat value chain, as well as to determine the knowledge and attitudes of those involved in the trade, particularly concerning food safety and public health. In this paper, we focus on Africa’s most populous megacity—Lagos, Nigeria—where only limited research on this subject has been conducted. The lack of empirical investigation into wild meat hygiene in this region means that the health risks posed by the Lagos wild meat value chain may be underappreciated. Hence, this study identifies the major hygiene and sanitary regulation challenges of a typical urban wild meat value chain, providing crucial data to support interventions that aim to improve global food safety, enhance environmental health, and strengthen hygiene policy enforcement in Lagos and other urban cities with similar challenges both in the Global South and other regions of the world.

## Materials and methods

### Study area

**Fig 1:**
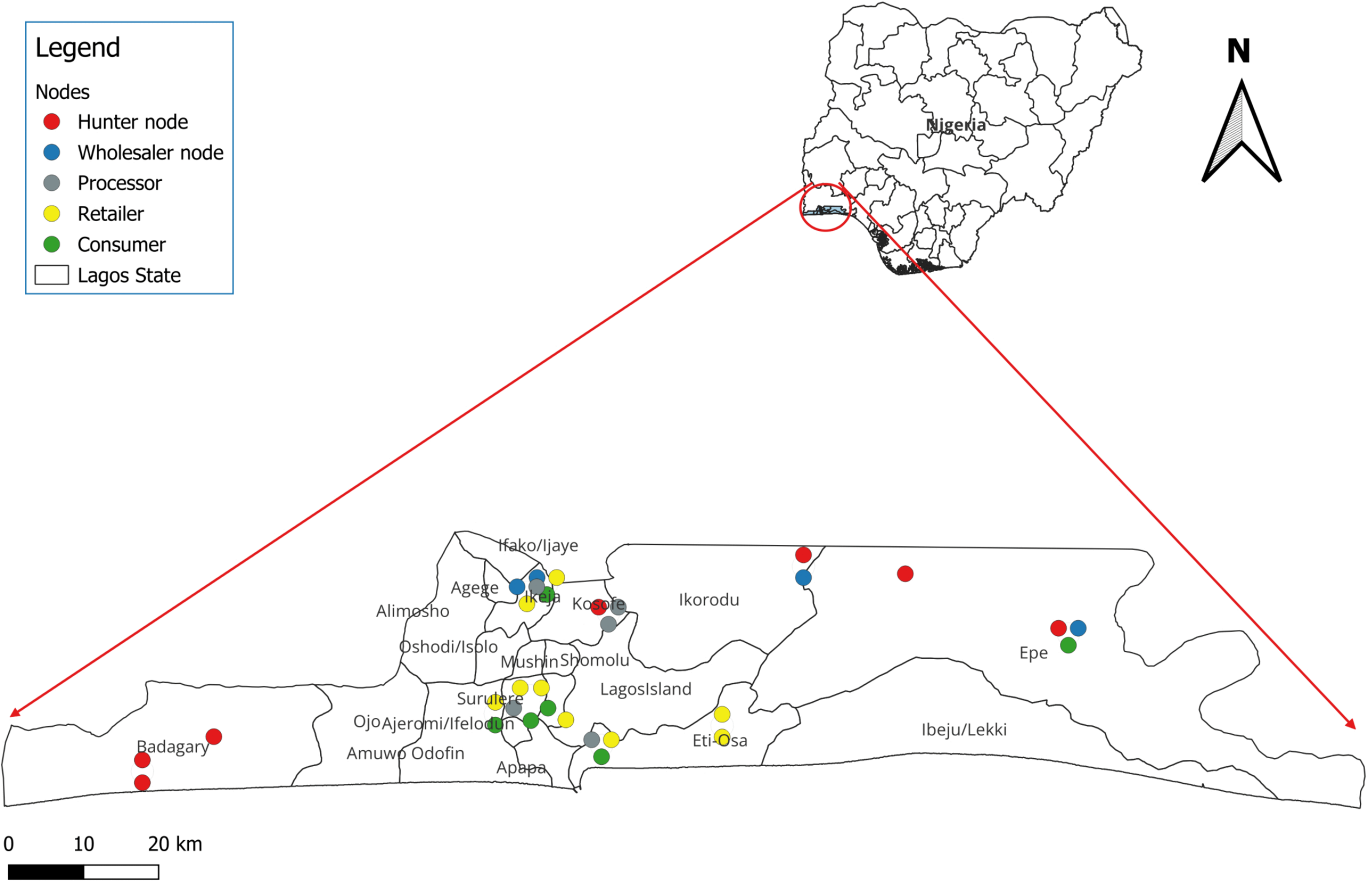
Map showing study sites and wild meat value chain nodes in Lagos (https://open.africa/dataset/shape-file-of-nigeria).

This study was conducted in Lagos, situated in southwestern Nigeria. Lagos’s dense population, estimated at 26 million people [21], supports a bustling center of commerce where wild meats are traded alongside other goods. Fig 1 presents a map of Lagos city showing the data collection sites.

### Ethical approval

Approval for this research was received from the National Health Research Ethics Committee (NHREC) of Nigeria, with approval number: NHREC/01/01/2007- 07/12/2022.

### Ethics statement

Human study participants were recruited from 22-01-2023 to 17-06-2023. Before every interview, the research team shared the project information with the participants through a Project Information Sheet (**S1 File**) and sought their consent to participate in the study (**S2 File**). We received verbal consent from all participants in this study. Each consent was documented by ticking on the consent form with the initials of each participant. All consents received were physically witnessed by the lead author. Additional information regarding the ethical, cultural, and scientific considerations specific to inclusivity in global research is included in the supporting information (**S1 Checklist**).

### Data collection

The data presented in this manuscript relates to items 8–18 of the interview guide (**S3 File**). Semi-structured interviews were conducted during a Lagos wild meat value chain mapping exercise. Participants were purposively selected based on their roles in the value chain. They were hunters (n = 8), wholesalers (n = 5), processors (n = 9), and retailers (n = 12). Although the consumer nodes were identified, no wild meat consumers agreed to participate in the study. With the aid of a local interpreter, interviews were conducted in the local “*yoruba*” language for participants who could not understand or speak English. All responses were recorded. We asked the participants about their roles in the value chain, wild meat regulations, and their hygiene practices. “Hygiene” in this study context was used to refer to the specific methods and practices used by individuals involved in the hunting, processing, transporting, storing, preservation, packaging, displaying and selling of wild meat that may impact its quality and safety. Participants were also asked about their perception of zoonoses, and if they believed wild meat may be a source of zoonotic and food-borne disease transmission to humans. To gain more insight into how to improve the hygiene situation, we sought to unravel the motivations behind the reported hygiene practices, using the transcribed data. This was mostly indicated in follow-up questions that had to do with “why” actors might do the described activities.

### Data analysis

Recordings from the interviews were transcribed verbatim into Microsoft Word and analyzed qualitatively by thematic analysis, using an inductive approach. The analysis process involved familiarization, coding, theme generation, reviewing, defining, and reporting [22]. The transcripts were read multiple times by the researchers to review any discrepancies between the audio recording and the written records of the responses from the participants. From the familiarization, the initial codes were arrived at using the study themes. From the initial codes, major codes emerged in the forms of words, phrases and sentences [23]. Systematically applying these codes to the entire dataset led to the discovery of patterns, connections, and recurring themes. This process aided in theme formation through the grouping of similar codes. Categories already covered were collapsed. Through this process the major codes evolved into themes. To ensure that the major themes captured the essence of the data, the initial themes were reviewed and refined by comparing them with the coded data. The themes that captured the essence of the data they represent were retained, while those that deviated were either refined, restructured, or merged (**S4 File**). We considered the factors (emergent codes) grouped under each of the three overarching themes as drivers of the hygiene practices of actors in the value chain**.** The overarching themes, codes, and their description are shown in Table 1.

**Table 1:** Codebook showing the emergent codes and their covering themes.

Major themes	Codes (sub-themes)	Description of the comments captured
Culture	Social norms	Cultural beliefs and perceptions
	Gender	Gender roles and biases
	Distrust	Disbelief in government-sponsored zoonotic awareness efforts
	Experience	History and personal experience of actors
Infrastructure	Preservation	Preservation methods and techniques for storing wild meats
	Transportation	Movements of wild meat from forests, and from actor to actor
	Water	Water availability for cleaning of slaughter premises and wild meat hygiene
	Display	Facilities for showcasing wild meats
Regulation	Formal regulation	Government regulations and the enforcement of hygiene and environmental sanitation
	Informal regulation	Laws and regulations within organized wild meat unions and self-governance

## Results

This study identified key issues for the wild meat value chain in Lagos, Nigeria, each presenting unique challenges as a result of social norms, practical constraints, and infrastructural challenges.

### Socio-demographic characteristics of study participants

Diverse but complementary value chain roles and activities were reported. This encompassed sourcing/production, processing, packaging, sales, and marketing. Table 2 shows participants’ demographics and roles in the Lagos wild meat value chain.

**Table 2:** Participants’ demography and roles in the value chain.

Participant category	Number participating	Gender	Age (years)	Education level	Daily activities	Main role
Hunters	8	M	51	PS	Hunt and capture wild animals from the forests; check traps set at agricultural farmlands; harvest wild meat	Sourcing of wild meat. Serve as the primary link between wildlife and the value chain.
		M	54	PS		
		M	45	PS		
		M	63	PS		
		M	73	IE		
		M	52	SS		
		M	62	IE		
		M	48	PS		
Wholesalers	5	F	35	SS	Receive or purchase whole carcasses from hunters or processed bulk wild meat from processors for resale. Sometimes, transport wild carcasses to markets and buyers.	Off-taking of freshly hunted wild meat; serving as an intermediary and distributor of large quantities of wild meat from forests to the city center.
		M	51	SS		
		M	54	PS		
		F	49	PS		
		M	47	TE		
Processors	9	F	45	SS	Receive or buy, clean, cut, cook, or roast fresh wild carcass; package wild meat for further local distribution or export.	Transformation and value addition to raw wild meat, thus contributing to meeting consumer preferences for processed products or specific cuts.
		F	52	PS		
		F	48	PS		
		F	41	SS		
		F	51	SS		
		F	58	SS		
		F	55	PS		
		F	44	PS		
		F	44	PS		
Retailers	12	M	37	PS	Brand, advertise, hawk, and trade wild meat.	The link between proximal and distal nodes; provides easy access to a variety of processed wild meat to satisfy consumer preferences.
		F	44	TE		
		F	56	SS		
		M	66	SS		
		F	31	PS		
		M	48	SS		
		M	40	SS		
		F	37	TE		
		F	37	SS		
		F	52	PS		
		M	50	PS		
		F	54	PS		

*PS-primary school; SS-secondary school; TE-tertiary education; IE-informal education.

Half of the study participants (50%, n = 34) had attained primary school education, 35.2% (n = 34) had secondary education, while only 8.8% (n = 34) attained tertiary education. The majority of participants were females (53%, n = 34), while males accounted for 47% (n = 34), predominating at the hunter node. The mean age of the participants was 48.4 years (SD = 4.86).

### Hygiene practices of actors in the Lagos wild meat value chain

Results of the thematic analysis revealed three overarching themes: (i) culture (ii) infrastructure, and (iii) regulation.

#### Culture.

Here, we describe the practices that were reported by participants or observed by the researchers during data collection, which we see as being explicitly linked to the wild meat actors’ hygiene. Four sub-themes emerged under this major theme. They were: social norms, gender, distrust, and experience.

**Social norms:** Cultural beliefs and perceptions dictated certain wild meat handling practices among actors. For example, actors did not prioritize hand washing with soap after handling fresh or processed wild meat, because they believed they were immune to infectious diseases:

*“Washing hands with soap after handling is not necessary because as black people, our immunity is very high, so we can’t be sick [through wildmeat]. Take the case of COVID-19 for example”* (KII012F, Wholesaler).

Another common belief was that wild animals were pure and hence could not transmit diseases:

*“………I don’t need to wash my hands after handling, because wildlife is pure. There is nothing like that kind of disease [zoonoses]”* (KII019F, Processor).

*“After handling, we rinse hands with whatever we are drinking, especially beer or palm wine. I hear it is even better than hand sanitizer”* (KII004M, Hunter).

Traditional hunting practices were also exhibited in the handling of wild meat. Fresh carcasses were carried on shoulders, while some were butchered in the forests:

*“Anytime we get [hunt] rodents, we put inside a sack bag; but for animals like antelope or deer, we carry it on our shoulders. I don’t know why, but that is how we do it.”* (KII001M, Hunter).

*“When I get wild animals, I butcher and roast it there on the farm before taking it home”* (KII007M, Hunter).

Field observation revealed the mixing of different species of wild meat. As a general practice, freshly killed animals of diverse species and sizes were mixed during transportation. Participants’ responses indicated that the local people liked the taste and aroma that some species give to other meats when mixed during cooking or storage:

*“After purchase, I put all the animals together into sacks, and transport them on my motorbike to the market for sales. That is how we do it here*” (KII009M, wholesaler)

*“I mix antelopes, porcupines and other animals [species] with snakes during cooking. The reason is that snakes give a delicious taste to the other meat, which many of my customers love”* (KII031M, retailer).

Similarly, actors referenced consumer preferences and past generations’ practices to justify their actions, as seen in the excerpt:

*“I mix different meats like rats and snakes while cooking. My customers have never complained, and I do not want to lose them”* (KII027F, retailer).

**Gender:** Gender roles were prevalent in the value chain, with certain tasks reserved for women. Hence, while men hunted or trapped wild meat, women were responsible for processing and preparing wild meat delicacies at markets, drinking bars, and homes:

*“As a farmer, I set traps in my farm, and sometimes I am lucky to catch some rats. When I return home, I usually give them to my wife to prepare for the whole family to enjoy”* (KII002M, hunter).

*“After hunting, I don’t process bushmeat. It is the duty of women. The only challenge is that sometimes they are few and before they finish, some animals [carcasses] start to rot because of the long time”* (KII005M, hunter)

Participants’ observation revealed that actors who were nursing mothers also juggled the task of caring for their young with wild meat processing.

**Distrust:** A significant gap in the knowledge of zoonotic disease transmission through wild meat was seen. Participants in their responses were dismissive of the health risks, citing a plot by the government to disrupt their livelihoods, as captured in the following excerpts:

*“I usually hear the government people talking about it. Let me tell you, there is nothing like zoonoses and we cannot contract any disease through handling wild meat”* (KII003M, Hunter).

*“That Ebola thing is a lie by government people, to discourage our business, and discourage people buying from us. Here, we don’t believe [such]. There is nothing like Ebola”* (KII008F, Processor).

**Experience:** The use of personal protective equipment (PPEs) such as gloves and face masks was not practiced among the actors. Results indicated that the attitudes and perceptions cultivated over time through the personal experiences of actors discouraged their use of protective clothing and equipment. For example, they believed that using gloves would suggest their wild meat products were unsafe, which they believe will send the wrong message to intending customers, as seen in the excerpt:

*“You should not be seen wearing hand gloves while holding wild meat. If you do, then you are telling the people that your wild meat is not safe, and they will not buy it”* (KII024F, Retailer).

“*We don’t use hand gloves. I have never seen anyone doing that. The only problem is that sometimes we get skin scratches from animals that are not fully dead. But it is normal for us”*. (KII008M, Hunter)

#### Infrastructure.

In this section, we describe particular practices that were reported by participants or observed by the researchers that could be explicitly linked to the process of wild meat harvesting, transport, and processing. The sub-themes that emerged here are: preservation, transportation, display, and water.

**Preservation:** The lack of refrigeration infrastructure and equipment for preserving and cooking wild meat during hunting expeditions also influenced the practices:

*“Sometimes, sales is poor and I still have meat which I don’t want to spoil [rot] and produce a bad smell. So, I remove the internal organs, to slow down the spoiling [spoilage]”* (KII010F, wholesaler).

*“While hunting, I sometimes eat stale or half-cooked meat. Because the forest is [may be] wet so the wood cannot burn to heat up or roast any meat very well”* (KII007M, hunter).

**Transportation:** Participants reported that during hunting expeditions, they slaughtered large animals such as buffalo in the forests, where sanitary hygiene is not guaranteed:

*“When we have large animals, we butcher it in the forest for easy transportation”* (KII006M, hunter).

Also, due to transportation constraints, meat from different species was mixed. Different body parts (e.g., hoofs, horns, intestines, and hides) were also mixed with meat cuts, increasing the risk of cross-contamination:

*“As you can see, I put all the animals inside my hunting bag; both grasscutter, hare, antelope, rats, and others. That way, it is easy for me to carry them out of the bush”* (KII001M, hunter).

**Water:** Field observations revealed that processors washed fresh wild carcasses with water drawn from artesian water wells, boreholes, nearby rivers, or rainwater collected in containers, depending on the availability of these sources. Others paid for traditional water vendors popularly called “*Mai ruwa*” to supply them with water sourced from farther places. Also, processors did not wash knives frequently due to the lack of water, as illustrated in the excerpt:

*“We don’t need to be washing our knives all the time. In this place that you see us, where will we get the water to do all that? We wash everything when we are done for the day”* (KII017F, processor).

**Display:** Field observations revealed that the lack of requisite facilities for safe display of processed products also hampered the hygiene standards. Wild meats were openly displayed in trays or hung with ropes on roadsides with heavy vehicular traffic and movements. Reason for this practice is seen in the excerpt:

*“I put out all my products in the open so that customers and people traveling can easily see it. If I had a showcase with glass, I would have used it…although that is not popular here”* (KII033F, retailer).

#### Regulation.

Here, we describe particular practices that were reported by participants or observed by the researchers that could be explicitly linked to the regulation of the value chain and actors’ activities.

**Formal regulation:** Participants’ responses indicated that there was a compulsory environmental sanitation by-law enacted by the government of Lagos State, to which all markets, stores, and business premises were subjected. The act, referred to as “Lagos State Environmental Management Protection Law 2017” was the only formal regulation that guided the practices of wild meat actors in Lagos. Part VI (section 188) of the enacted law, which established the Lagos State Environmental Protection Agency [24], states:

“*Housing estates, hotels, commercial facilities, waste management facilities, hospitals, abattoirs and livestock shall not discharge or cause to be discharged any trade and industrial effluent into the public drain or natural environment without a permit from the Agency. (2) Effluent discharged under this section shall not exceed the permissible limits/levels contained in the Regulation of the Agency*”.

Within main city centers, environmental sanitation officers made rounds to inspect facilities such as markets, restaurants, and shops. Levies were also collected for environmental sanitation and waste collection, with fines placed as punishment for non-compliance:

*“…… We have cleaning of our market every Thursday morning, and it is compulsory for all of us. On that day, we can only open our business at 10 a.m.”* (KII0026F, retailer)

*“Although anybody can clean at any time, our general cleaning is on Thursday, and it is always a must [compulsory]. Anybody that does not do it will pay a fine, which sometimes is heavy. But that is only when the government officers come for inspection of the environment”* (KII0015F, processor).

Participants were not aware of any other formal regulations on wild meat hygiene and safety, outside the routine weekly environmental sanitation. In their view, participants believed that the inspections were ineffective, mainly focused on the city centers, and the collection of fines from defaulting traders. Hence, while the general cleaning exercise may be carried out wholeheartedly by some, some participants expressed that they cleaned their work environment out of compulsion, and to evade payment of non-compliance fines:

*“The issue of cleaning every Thursday is not really for us. It is general law for all the shops and markets in Lagos. I only joined because I am part of the market. Sometimes we don’t clean, we just close business till the cleaning time is over”* (KII009F, wholesaler).

No participant reported veterinary inspection of their fresh or processed wild meat. Also, no meat inspectors were seen in the wildlife markets, processing, and retail points that were visited by the research team.

**Informal regulation:** Participants reported that there were unwritten codes of conduct guiding the wild meat association members’ activities. The wild meat association is an informal body or union comprising wild meat hunters, wholesalers, processors, and retailers in an area or community. This body is often formed and governed by members elected from amongst the wild meat value chain actors. They also form their informal regulations or codes of conduct. According to participants’ responses, only a few of these codes of conduct addressed hygiene issues, as seen from the excerpts:

*“We have laws that we create and run by ourselves. But they are mostly about the business side. The one that has to do with the meat is that no one should process or sell rotten meat”* (KII016F, processor).

*“Any dried or roasted meat remaining from the previous day must be heated before sale on the next day. Also, no one must sell meat from an animal that died by car accident”* (KII030F, retailer).

Some participants (n = 7) reported that they did not belong to any market union, did not attend any wild meat association meetings, nor could be held accountable by any established codes of conduct. Responses also indicated that in some areas, those selected for monitoring and enforcement of the informal regulations were also actors themselves in the value chain, who were often too busy with their personal businesses and schedules. As a result of this lack of oversight, wild meat from road kills and animals that died from unknown causes was occasionally supplied and processed for sale. Also, compliance with regulations was sustained by trust and the social norm that a good conscience may guide members to abide by the codes of conduct, as seen in the excerpt:

*“Here, we believe in conscience. There is no reason to monitor anybody. Our members know the right thing to do, and are judged by their conscience, good or bad”* (KII013M, wholesaler).

## Discussion and conclusions

Wild meat value chains continue to constitute a platform for foodborne and zoonotic pathogen transmission and spread in urban cities. In this study, we used semi-structured interviews and participants’ observation to gain insights into the hygiene knowledge and practices of actors in the Lagos wild meat value chain. Grouping our findings into three overarching themes (actors’ personal hygiene, wild meat process hygiene, and governance), we identified cultural influences, shortage of infrastructure, and poor regulation as the main drivers of poor hygiene practices of actors in the wild meat value chain.

### Cultural influences

Our study results reveal that actors culturally associated the use of gloves with a stigma, hence did not prioritize washing of hands before and after handling wild meat. Hand hygiene is a critical factor in the safety of final meat products. Studies [25,26] show that bare-hand contact with meat can introduce foodborne pathogens (e.g., *Salmonella* spp., *Campylobacter* spp., *Listeria* spp., *Escherichia coli*, etc.), most of which have low infective doses. Therefore, hand washing and the use of gloves have been outlined as the main methods for reducing bacterial cross-contamination from hands to ready-to-eat food [27]. While there is no documented evidence of the harm of hand washing with palm wine, beer, or other alcoholic drinks, the practice is inappropriate and a breach of established food safety standards. According to the United States Food and Drug Administration [28], food safety standards aimed at eliminating harmful bacteria and preventing contamination require handwashing with soap and water and then waiting for at least 20 seconds before handling food. Beer and palm wine are not effective cleaning agents, as they do not have the necessary friction to remove dirt, their alcohol is not strong enough to effectively destroy bacteria [29], and they could be contaminated with pathogenic microorganisms [30]. We posit that these poor personal hygiene practices may also be associated with the low literacy and awareness levels of actors, as seen in the demography results, where only 50% actors had obtained primary education (Table 2). Ultimately, consumers are the main victims of health risks related to poor personal hygiene, as it can lead to food poisoning resulting from microbial contamination [31]. Furthermore, our analysis suggests that certain unhygienic practices, such as “*carrying wild meat on the shoulders*”, were borne out of age-long cultural practices regarded as norms by actors [19,32]. Social norms not only hindered the adoption of proper hygiene practices but also sustained the risky behaviors of the actors. Ancient traditional social norms used for hunters’ team cohesion during group hunting (e.g., the butchering and sharing of meat in the forests) [19], ensure that unsanitary practices are maintained, predisposing wild meat to contamination. The hunters are also exposed to zoonotic risks through blood and aerosols from potentially infected wild meat. However, while the unsanitary practice of direct bodily contact with wild meat may be influenced by cultural norms, we argue that such practices may also be borne out of necessity, since they occur in environments that lack the necessary sanitation. This highlights the practical difficulties in ensuring wild meat hygiene and safety [18]. Furthermore, gender bias emerged as a critical barrier to achieving overall hygiene conditions, as men declined participation in cleaning and processing activities. According to a report [33], the highest risks of disease transmission in wild meat value chains occur at the processing stage (cutting, splitting, skinning, etc.). With the higher involvement of women at the processing stage, as seen in the socio-demography results (Table 2), this could mean that women were placed at a higher risk of contracting zoonotic infections from potentially infected carcasses. Participants’ responses also pointed to public distrust in the government, with actors having negative cultural perceptions about zoonotic risks. Similar to our finding, people rejected the health messages linking wild meats to Monkeypox in Nigeria [34]. Also, informants in Guinea cited political events as the rationale for the ban on wild meat trade during the outbreak of Ebola virus disease (EVD) in 2016 [35].

Awareness and education of actors is key in improving wild meat hygiene and mitigating health risks of wild meat [14,36,37]. Consequently, educational campaigns should target all nodes and actors within the value chain to shift their attitudes and perceptions towards gender roles, public health information, and safer wild meat practices. Also, for wild meat hygiene interventions to be effective, local cultural considerations must be integrated [38]. Hence, relevant global and local bodies such as policy makers and governments at all levels should implement culturally-sensitive orientation programs to raise awareness about the zoonotic and food-borne disease risks associated with poor hygiene practices. Such programs should also be participatory [37], involving community, religious. and traditional leaders, to ensure the message resonates with the social norms, cultural beliefs, and practices of their communities.

### Infrastructure deficits

The lack of requisite infrastructure significantly influenced wild meat hygiene. Meat spoilage is often caused by a lack of storage facilities and unfavorable ambient temperatures [39]. Storage of meat at 4°C within 4 hours post-slaughter, or freezing at -20°C, is necessary to slow down the microbial growth of spoilage bacteria, ensuring that meat is preserved [40]. However, many countries in sub-Saharan Africa mostly rely on primitive methods of meat preservation, due to the absence of modern refrigeration facilities and electricity [15]. Over 760 million people worldwide lack access to electricity, especially in rural communities of South Asia and sub-Saharan Africa [41]. This compelled the actors to resort to rudimentary methods of wild meat preservation, such as the use of slow-burning firewood. Significantly, it is observed here that this traditional method, though resourceful and meeting consumer preferences, does not adequately prevent microbial growth. Also, the infrequent washing of knives and other tools arising from the lack of consistent access to potable water contributes to potential cross-contamination and the proliferation of pathogens in wild meat. Hence, the lack of refrigeration facilities, electricity, and potable water contributed to poor wild meat hygiene practices, especially at the processor node, where transformation and value addition to raw wild meat occur along the chain (Table 2).

The provision of hygiene infrastructure and preservation facilities can offer great marginal benefits to public and occupational health [42]. Therefore, we recommend that clean water access be provided for all actors in wild meat value chains to facilitate proper personal and wild meat process hygiene. Also, investments must be made in modern cold room facilities and power supply to reduce spoilage and microbial contamination of wild meats.

### Poor regulation

In this study, there was no report or observation of meat inspection, inferring wild meats were not assessed for their quality and safety pre- and post-slaughter. Aligning with our findings, another study [43] reported that there was no veterinary inspection of wild meats in many African countries, despite their well-known and established wild meat markets and traditions [11,14,44]. However, there is a shortage of meat inspection officers in many African nations [46]. We posit that this could be due to several factors, including under-resourcing, under-financing, and neglect. The lack of veterinary inspection of wild meats continues to exacerbate the use of unhygienic handling and processing techniques, thereby exposing wild meat actors and the public to health risks. Additionally, our study highlights that compliance with formal regulations was only practicable within urban organized markets; hence, regulations guiding the practice of actors in the peri-urban areas were mostly informally regulated. However, informal regulations derived from social norms and sustained through trust were not sufficient to address the hygiene challenges. In general, the regulatory framework governing wild meat trade in Nigeria borders on the Endangered Species (Control of International Trade and Traffic) Act [47], and the National Environmental (Protection of Endangered Species in International Trade) Regulations [48], in addition to international agreements such as the Convention on International Trade in Endangered Species of Wild Fauna and Flora (CITES) [49]. However, the findings of this study and a previous report [19] indicate that the regulatory landscape governing the wild meat trade in the study area is marked by insufficient regulatory enforcement, widespread non-compliance, and economic factors. The enforcement of wildlife and food safety regulations in Nigeria is frequently undermined by corruption [20,44], which may explain the difficulties in regulating the wild meat value chain. Hence, while the poor hygiene practices in the value chain may have been driven by the lack of awareness, social norms, gender roles, and infrastructural deficits, they were majorly exacerbated by weak regulatory enforcement. Strengthening the enforcement of existing regulations can be achieved through increased and equitable funding of relevant continental, regional, and national food safety regulation and enforcement agencies. Air and land border veterinary quarantine services should be strengthened and equipped to ensure only wild meats that meet globally accepted sanitary standards are allowed passage. Standardized hygiene practices and guidelines [45,50] for all actors should also be implemented to reduce food safety and public health risks in the wild meat value chain.

### Limitations to the study

Many identified wild meat actors, especially consumers, declined participation in the study, citing fear of publicity and potential adverse outcomes for their trade. Hence, we conducted in-depth interviews with a sample size of thirty-four (n = 34) study participants who gave consent, which created a limitation in carrying out a quantitative analysis of results. Also, due to the sensitivity of the study subject and lack of written permission from the study participants, some study data such as photos, which could aid the analysis and better understanding of our study results, could not be used.

## Supporting information

S1 File Project information sheet. (DOCX)

S2 File Informed consent form. (DOCX)

S3 File Interview guide. (DOCX)

S4 File Thematic data. (XLSX)

S1 Checklist Inclusivity in global research. (DOCX)
