# Supplementary material for: Hygiene knowledge and practices in the Lagos wild meat value chain: Cultural influences, regulatory gaps, and infrastructure needs
Source: PLOS Glob Public Health. 2026 Jan 16;6(1):e0004321. doi: 10.1371/journal.pgph.0004321 (PMC12810893; doi:10.1371/journal.pgph.0004321)
Supplement: S2 File — (DOCX) [file pgph.0004321.s002.docx]

**Declaration of Consent**

I have read the project information sheet or received information on this study and decided that I will participate in the research project. Its general purposes, the particulars of involvement and possible risks and inconveniences have been explained to my satisfaction. I understand that I do not have to participate in the study and can withdraw at any time. My signature also indicates that I have received a copy of this consent form. Each page of the consent form is initiated by me and the study staff, to indicate that the study staff has reviewed all of the pages with me.

*By ticking each box, you are consenting to the elements of this study. It will be assumed that unticked box means that you DO NOT consent to that part of the study, and this may make you ineligible to participate in the study.*

I agree to allow my initials on each page of the consent form.

There might be other research questions that investigators could study in the future with the help of samples like the one collected from the animals.

I agree to fill the questionnaire, or take part in interview and discussions.

I understand I will not be re-contacted about future potential use.

I understand that my participation is voluntary and that I do not have to take part if I do not want to. I understand that I am free to withdraw from this study if I change my mind.

____________________________________________ _____________

Subject name and signature Date

____________________________________________ _____________

Parent/Legally Authorized Representative (if applicable) Date

I am unable to read but this consent document has been read and explained to me by ___________________ (name of reader). I therefore volunteer to participate in this research.

____________________________________________ _____________

Subject name and signature Date

____________________________________________ _____________

Witness Date

# Signature of Investigator or Responsible Individual:

To the best of my ability, I __________________________(name of investigator / person obtaining consent) have explained and discussed the full contents of the study, including all information contained in this consent form, and I have answered all questions from the research subjects and those of his/her parent(s) or legal guardian.

____________________________________________ _____________

Signature Date
