# Supplementary material for: Hygiene knowledge and practices in the Lagos wild meat value chain: Cultural influences, regulatory gaps, and infrastructure needs
Source: PLOS Glob Public Health. 2026 Jan 16;6(1):e0004321. doi: 10.1371/journal.pgph.0004321 (PMC12810893; doi:10.1371/journal.pgph.0004321)
Supplement: S3 File — (DOCX) [file pgph.0004321.s003.docx]

**Supplement 1: Interview Guide**

1. What is/are your role(s) in the value chain?
2. From where/whom do you obtain the wild meat?
3. In what state do you obtain the wild meat (fresh whole carcass, processed, etc)
4. To whom do you supply your products?
5. Are there any specific practices you engage in?
6. Is there a group or association for wildmeat hunters or sellers in your area/market/community? If yes, are you a member?
7. Who are the leaders? (Kindly describe the leadership structure of the group)
8. How do you govern yourselves, and are there any regulations or codes-of-conduct that guide your activities or practices?
9. If yes, what are the regulations or codes-of-conduct?
10. Are there any punishments for defaulters? [*Ask for any examples of punishments*]
11. Do you wear PPA while handling fresh or processed wildmeat? [*Ask for reasons for any given answer*]
12. How do you transport your fresh carcass or processed wild meat products? [*Find out why he/she uses the particular method*]
13. How do you store your products? [*Ask to describe the method*]
14. How do you preserve your wildmeat or fresh carcass from spoilage?
15. Do you mix different species? At what point do you mix, and why?
16. Do you think that diseases that can be transmitted to humans through wildmeat? [*Follow any interesting leads and interrogate further*]
17. Can poor hunting or handling practices lead to transmission of diseases from wild meat to humans? [*Ask the reasons for any answers given*]
18. Do you wash your hands with soap before or after handling wild meat? [*if yes, ‘how’? If no, ‘why’*]

*Probe for any other related information*

THANK YOU FOR YOUR TIME
